# Assessing the Performance of a Noninvasive Glucose Monitor in People with Type 2 Diabetes with Different Demographic Profiles

**DOI:** 10.1155/2017/4393497

**Published:** 2017-12-20

**Authors:** Karnit Bahartan, Keren Horman, Avner Gal, Andrew Drexler, Yulia Mayzel, Tamar Lin

**Affiliations:** ^1^Integrity Applications Ltd., 19 Hayahalomim St., 7760049 Ashdod, Israel; ^2^Division of Endocrinology, Diabetes and Hypertension, David Geffen School of Medicine, University of California, 10833 Le Conte Ave., Los Angeles, CA 90095, USA

## Abstract

**Background:**

Noninvasive glucose-monitoring devices represent an exciting frontier in diabetes research. GlucoTrack® is a noninvasive device that indirectly measures glucose fluctuation in the earlobe tissue. However, GlucoTrack measurements may be susceptible to effects of quasi-stable factors that may be affected by demographic profiles. The current study, thus, examined device performances in people with type 2 diabetes with different demographic profiles, focusing on age, gender, body mass, and whether the earlobe is pierced.

**Materials and Methods:**

Clinical trials were conducted on 172 type 2 adult diabetic subjects. Device performance was clinically evaluated using the Clarke error grid (CEG) analysis and statistically assessed using absolute relative difference (ARD).

**Results:**

CEG analysis revealed that 97.6% of glucose readings were within the clinically acceptable CEG A + B zones. Mean and median ARD were 22.3% and 18.8%, respectively. Likelihood ratio and parametric bootstrap tests revealed that there were no significant differences in ARD values across age, gender, body mass, and whether the earlobe was pierced, indicating that the accuracy of GlucoTrack remains consistent across the tested demographic profiles.

**Conclusions:**

Our results suggest that GlucoTrack performance does not depend on demographic profiles of its users and it is thus suitable for various people with type 2 diabetes.

## 1. Introduction

Diabetes is a chronic metabolic disorder in which blood glucose levels fluctuate outside the normal range. It has become a worldwide epidemic with about 415 million people worldwide diagnosed with diabetes in 2015 [1]. The burden of diabetes is enormous, as it imposes an excessively high human, social, and economic impact on individuals, countries, and national health systems. The lion-share of the burden is associated with diabetes-related complications, which may lead to morbidity, disability, decline in quality of life, and premature mortality [2, 3].

Abundant evidence demonstrates that diabetes-related complications can be prevented or delayed by maintaining tight glycemic control [4–6]. Self-monitoring of blood glucose (SMBG) was shown to be a vital component in achieving this goal [7–9]. SMBG is required as part of self-management and ongoing education for treatment and is assumed to improve adherence to pharmacological treatment and motivate patients to make appropriate lifestyle changes [7, 10]. In particular, it is useful in obtaining information about individual glucose profiles, as well as helping to understand the effect of medications and one's habits, including exercise and food intake, on glucose profiles.

However, commercially available devices for glucose measurement are invasive, leading to low SMBG compliance, especially among people with type 2 diabetes, due to the painful skin lancing and complex test procedures [11, 12]. Therefore, considerable efforts have been attempted over the last few decades to develop noninvasive (NI) devices that promote more frequent self-glucose monitoring [13–15].

**Figure 1:**
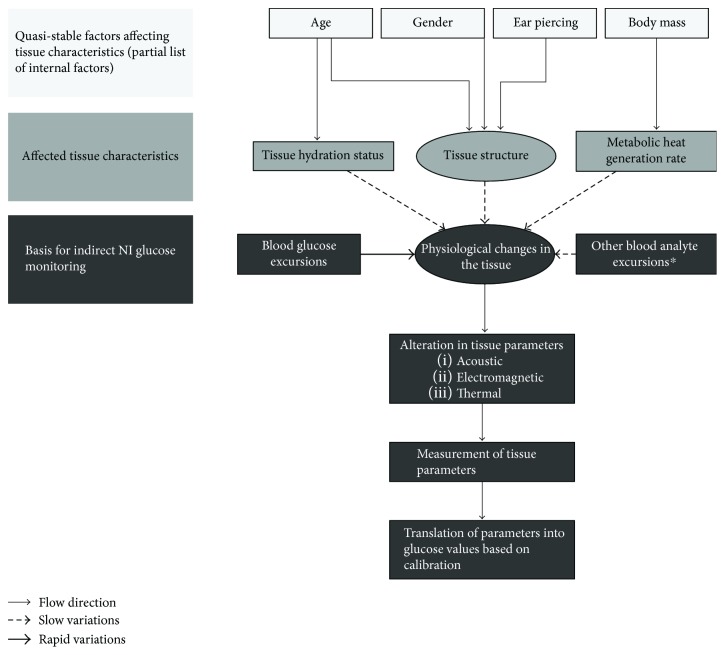
Schematic description of the quasi-stable factors' effects upon the NI measurement by GlucoTrack. A partial list of quasi-stable factors affecting tissue characteristics (light gray shapes), affected tissue characteristics (dark gray shapes), and the measured tissue parameters with their effect on GlucoTrack technologies (dim gray shapes). Thin solid arrows represent flow direction, dashed arrows represent slow changes, and thick solid arrows represent rapid changes. ^∗^Blood analytes with slow variation relative to glucose.

GlucoTrack (Integrity Applications Ltd.) is a NI glucose-monitoring device [16, 17]. Device's principle of operation is based on tracking the physiological effects of glucose variations in the earlobe tissue using three independent technologies: ultrasonic, electromagnetic, and thermal. The device measures specific ultrasonic, electromagnetic, and thermal parameters of the tissue, which occur due to glucose-related shifts in ion concentration, density, compressibility, and hydration of both cellular and extracellular compartments of the tissue [16, 17]. However, the measured tissue parameters may also be affected by factors other than glucose. These factors are of two types: those inducing slow to near-constant changes (i.e., quasi-stable factors) and those inducing relatively fast changes in tissue parameters (Figure 1). The effects of relatively fast changes are at least partially minimized through the use of a proprietary algorithm that combines three independent technologies' readings and calculates their weighted average [16, 17]. The current study focused on investigating the effects of quasi-stable factors, particularly those related to demographic profiles, on device performance. Notably, demographic profile affects tissue characteristics in a slow to near-constant manner.

For example, tissue structure and hydration status depend on age; older subjects may show reduced skin thickness and loss of water content [18–21]. Tissue characteristics may also be gender-related with men's skin being generally thicker than women's [22]. Additionally, earlobe piercing produces a scar tissue, which is rich in collagen and thus may alter tissue contents [23]. Finally, metabolic heat generation is affected by body mass [24] (Figure 1). The present study, thus, aimed to assess the performance of GlucoTrack among people with type 2 diabetes, focusing on the demographic categories of age, gender, body mass, and presence or absence of ear piercing.

## 2. Materials and Methods

### 2.1. Participants

242 diabetic subjects with type 1 or type 2 diabetes were screened. 40 subjects did not complete the clinical trial, and all type 1 subjects (i.e., 30 subjects) were excluded from this study according to the declared intended users of the device. Thus, the present study evaluated GlucoTrack performance on 172 type 2 diabetic subjects above the age of 18. 93 subjects were only on oral medication (54%), 9 subjects were only on insulin (5%), 58 subjects were on both insulin and oral medication (34%), and 12 subjects were not on either oral or insulin treatments (7%). Age, gender, body mass, and single ear piercing were chosen to represent the quasi-stable factors that may affect the measured tissue parameters. Subjects' demographic categorization is summarized in Table 1. Age subgroups were stratified as was previously done by Zoungas et al. [25]. Age ranged from 21 to 88 years. Body mass categorization included weight ranges that are equivalent to BMI < 25 kg/m^2^ (normal weight), 25 kg/m^2^ < BMI < 30 kg/m^2^ (overweight), and BMI > 30 kg/m^2^ (obese) for individuals of average stature (1.73 meters in Israel) [26].

### 2.2. Clinical Trials

Clinical trials were conducted in the diabetes unit of the Soroka University Medical Center, Be'er Sheva, Israel. The study protocol was approved by the local ethics committee and all participants signed an informed consent form.

**Figure 2:**
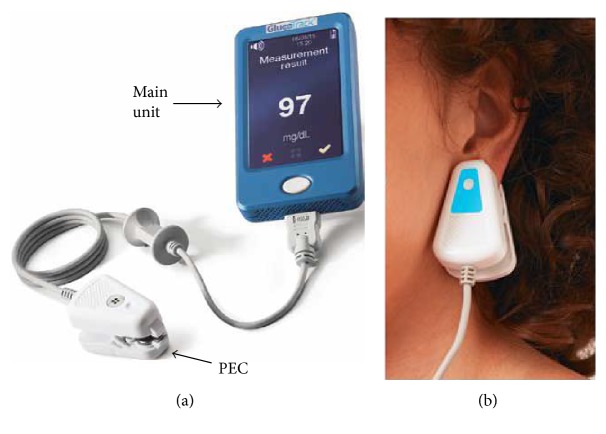
GlucoTrack NI monitoring device. (a) The device includes a main unit and three different sensor pairs, one per each of the three technologies, and all located at the tip of a personal ear clip (PEC). (b) Illustration of glucose measurement performance using GlucoTrack. The PEC is clipped to the earlobe for spot measurement.

Exclusion criteria included any condition that may hamper the contact between the personal ear clip (PEC; Figure 2(a)) and the earlobe, such as scratches, birthmarks, and multiple piercing. Participants receiving dialysis, as well as pregnant and nursing women, were excluded because of the imbalance in their water and mineral state [27, 28]. Type 1 diabetes subjects were excluded from the study since they are not included in the device intended use population. Due to constrains originated from the mechanical shape and size of the sensors' assembly, subjects with earlobes of less than 14 mm or above 25 mm in diameter and subjects with earlobe thickness lower than 3 mm or above 6 mm were also excluded from the study.

At the beginning of the trial, PECs were adjusted individually to the participants' earlobes for optimal fit, to ensure good and comfortable sensor-to-tissue contact for each ear width. Following adjustment, the PEC was calibrated for each patient to establish an individual baseline for the detection of physiological changes. Calibration involved three paired measurements of GlucoTrack and an invasive reference, with 10-minute intervals between each pair. The invasive blood glucose measurements were obtained from finger capillary blood using the HemoCue® Glucose 201 RT system (Ängleholm, Sweden).

Spot measurements using GlucoTrack were conducted by placing the PEC on the participants' earlobe for about 1 minute (Figure 2(b)). After completing the measurement, the ear clip was removed, and the glucose level was displayed on the screen of the device and recorded in the clinical research form.

The study involved two to three nonconsecutive days of sampling in the course of one month. Each trial day continued for 8 to 10 hours (between 8:00 AM to 6:00 PM) and included about 16 simultaneous paired measurement with GlucoTrack and HemoCue. On each trial day, subjects received meals and snacks in order to produce variability in their glucose profiles.

Trial day timeline was conducted as follows: the first paired GlucoTrack-HemoCue measurements were conducted in the morning following a night fasting. Measurements 2–6 were performed right after breakfast with 30-minute intervals between each pair. Next, participants ate one fruit followed by measurements 7 and 8, with 30-minute intervals in between. Measurements 9–16 were conducted right after lunch with 30-minute intervals. Between measurements 11 and 12, participants were offered an optional fruit dish [29].

### 2.3. Evaluation Methods

GlucoTrack performance was evaluated using clinical and statistical methods. Total and demographically stratified clinical performance of the device was evaluated using Clarke error grid (CEG) analysis [30]. Mean and median absolute relative difference (ARD) of paired GlucoTrack-HemoCue measurement readings was used to gain statistical insights on the device's performance across gender, age, body mass, and ear piercing. ARD was calculated as follows: ARD = |GlucoTrack-HemoCue|/HemoCue∗100[%] where GlucoTrack refers to the measurement result of GlucoTrack and HemoCue refers to the measurement result of HemoCue.

### 2.4. Statistical Analysis of ARD Values

An adequate statistical evaluation requires consideration of the nested nature of the data (data for each subject are organized in multiple levels) and residual distribution of the outcome (ARD). Hence, statistical framework of generalized linear mixed effects models was used to identify the model with the best fit to the data. The Akaike information criterion (AIC) was used to choose the best model that fits the data: gamma residual distribution with log link function. Repeated measurements were nested within corresponding days and the latter were nested within subjects. A fixed effect of the studied variables was defined and analyzed in R software (version 3.2.3) using lme4 package [31]. To assess the statistical effects of the tested demographic parameters on device performance, two tests were employed on ARD values: likelihood ratio test (LRT) and parametric bootstrap test (PBT) [32]. Both statistical tests were used in order to ensure the robustness of the findings.

## 3. Results

The clinical performance tests of GlucoTrack on 172 type 2 diabetes subjects demonstrated that 97.6% of glucose readings were within the CEG clinically acceptable A + B zones, with 52.9% in the clinically accurate zone A. Total mean ARD was 22.3% and total median ARD was 18.8%.

**Figure 3:**
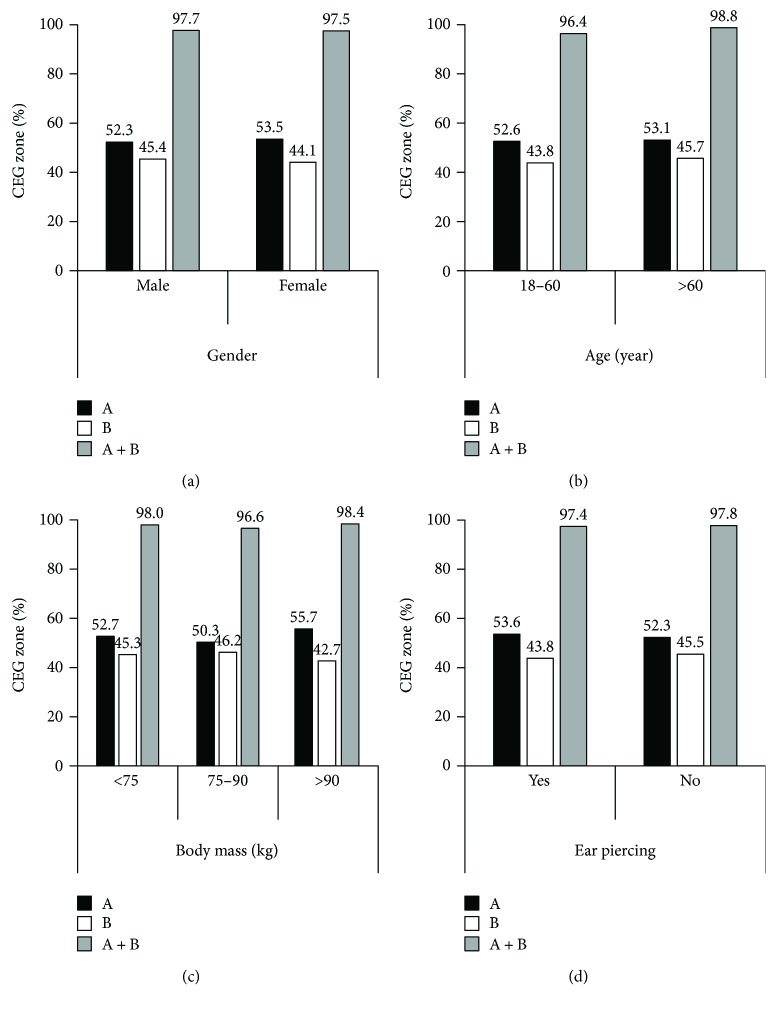
Clinical accuracy as a function of (a) gender, (b) age, (c) body mass, and (d) ear piercing.

**Figure 4:**
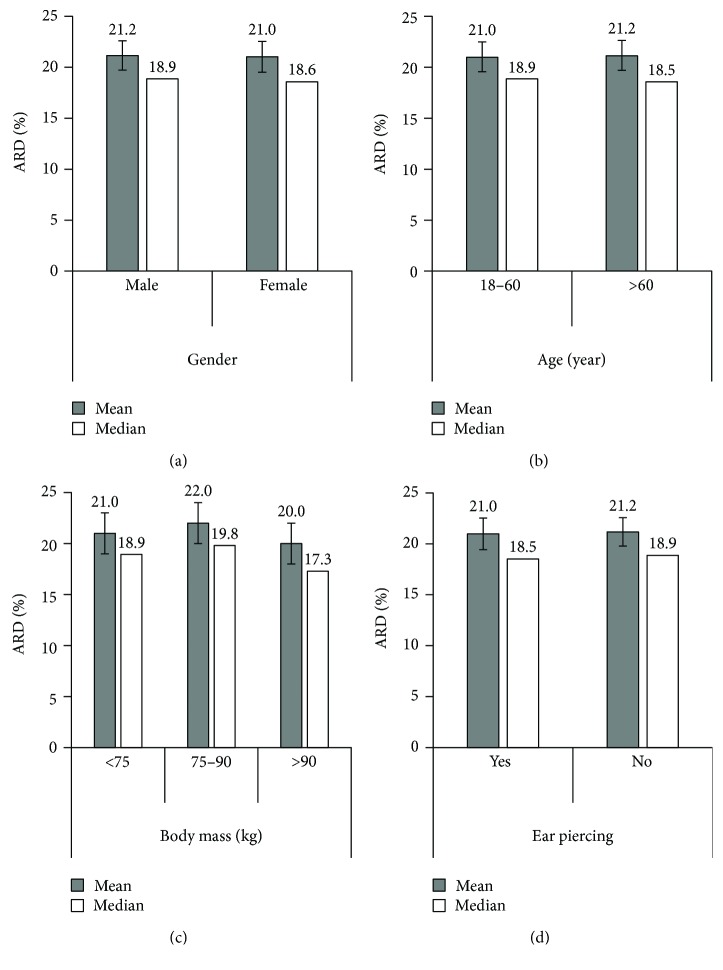
Numerical accuracy as a function of (a) gender, (b) age, (c) body mass, and (d) ear piercing. Mean ARD and its model-based upper and lower 95% confidence intervals and median ARD.

Between 2260 and 4382 paired GlucoTrack-HemoCue readings were obtained for each demographic category (Table 1). The categorical distribution of the measured values revealed similar patterns for the CEG A and B clinically acceptable zones, as shown in Figure 3. Comparison of ARD values within each demographic category revealed similar mean and median values (Figure 4). According to the LRT and PBT tests applied on ARD values, no significant differences were found between males and females (*χ* ^2^ (1) = 0.01, pLRT = 0.90, pPBT = 0.95) or between age groups (18–60 and over 60 years old): (χ^2^ (1) = 0.02, pLRT = 0.87, pPBT = 0.92). Similarly, neither statistical test found significant differences between body mass groups: (χ^2^ (2) = 2.69, pLRT = 0.26, pPBT = 0.22) nor between subjects with or without ear piercing: (χ^2^ (1) = 0.04, pLRT = 0.85, pPBT = 0.84).

## 4. Discussion

SMBG has an important role in diabetes management [33]. Recent attempts to promote self-monitoring of glucose include the development of NI devices [13–15], which may alleviate the pain associated with the frequent skin pricking. An example for such a device is GlucoTrack [16, 17], intended for people with type 2 diabetes or prediabetes. In order to reach high efficacy, such device should be applicable and suitable for a variety of users in terms of performance consistency. The current work aimed to evaluate the performance of GlucoTrack among people with type 2 diabetes with different demographic profiles, which may affect tissue parameters measured by the device. To this end, the effects of gender, age, body mass, and the presence of a single ear piercing on device performance were assessed in 172 people with type 2 diabetes. Generally, our results show that the accuracy of GlucoTrack does not depend on these factors.

Age, gender, body mass, and the presence of ear piercing may have effects on tissue characteristics and therefore on GlucoTrack performance [19, 23] (Figure 1). Previous studies have shown that men have thicker skin than women [22] and that ear piercing produces a collagen-rich scar tissue that is denser than a regular tissue [23]. Nevertheless, our results show that gender and pierced ears do not influence device performance. These results were found with respect to a clinical evaluation presented in CEG A + B zones and statistical analysis on ARD values (Figures 3(a), 3(d), 4(a), and 4(d)), signifying the robustness of the effect.

The demographic factor of age has been related both to tissue water content [18, 21] and to tissue thickness [19, 20]. Water content may influence device performance by directly affecting the thermal, ultrasonic, and electromagnetic properties of the tissue [23, 34] measured by GlucoTrack. Although the clinical accuracy of glucose readings in subjects under the age of 60 was slightly lower than that of older subjects (96.4% and 98.8% in CEG A + B zones, resp.), mean and median ARD values were similar (Figure 4(b)). Therefore, our findings suggest that age has no statistically significant influence on GlucoTrack performance.

The effect of body mass on metabolic heat generation [24] may also affect device performance since the rate of metabolic heat generation may influence several thermal properties within the tissue. There was a slight reduction in the clinical accuracy of GlucoTrack in the 75–90 kg body mass group relative to the other groups; the percentage of CEG A + B zones for subjects with lower or higher body mass (below 75 kg and over 90 kg) was 98.0% and 98.4%, respectively, as opposed to 96.6% for the 75–90 kg body mass group. However, mean and median ARD values were similar (Figure 4(c)), suggesting that body mass has no significant influence on device performance.

Overall, device performance was consistent in all studied demographic categories, indicating that its accuracy is similar for a variety of people with type 2 diabetes. We presume that the consistency in device performance originates from efficient individual calibration, which establishes a baseline for physiological change detection that is not expected to change substantially in the 6 months of device calibration period. It should, however, be noted that the performance of GlucoTrack is inferior to that of current invasive and minimally invasive methods, mainly due to the indirect nature of the measurement that subjects NI devices to suffer from a relatively low signal-to-noise ratio. For this reason, currently GlucoTrack should not be used for diagnosis and medication intake or treatment decisions should not be based only on measurements obtained by it. Nonetheless, the results of this study may significantly contribute to the emerging research of noninvasive glucose-monitoring devices and provide a milestone to this field.

There are several limitations to this study. First, GlucoTrack results were compared against HemoCue, rather than comparing them against gold standard reference samples. This, however, should not affect the interpretation of our results. GlucoTrack glucose reading is based on physiological effects occurring in the tissue as a whole, which may be subject to influences from additional factors. For example, factors affecting the time lag between interstitial fluid (ISF) and blood glucose concentrations may also affect measured tissue parameters and consequently device accuracy. One such factor is blood perfusion, which influences microvascular permeability and consequently may affect the physiological time lag between blood and ISF glucose levels [35–37]. Conditions that have been suggested to cause perfusion problems include the duration of diabetes, HbA1c levels greater than 7.5% [38], cardiovascular and renal disease [39], and smoking history [40–42]. Future research should address the effects of these factors on GlucoTrack performance. In addition, further studies should test the device in other populations in other countries. Nonetheless, it should be noted that the Israeli population is diverse in terms of skin tones and origin of birth (e.g., Europe, North and South Africa, Middle East, United States, and Asia), so that this clinical trial did include participants from various origins and skin tones. However, these could not compose statistically representative groups, since Israel has a limited number of individuals from these groups that are eligible for our studies.

In sum, GlucoTrack is suitable for people with type 2 diabetes with diverse demographic profiles. The unique glucose-monitoring device offers a noninvasive, painless, cost-effective, and simple way of self-monitoring glucose levels. We believe that the device will encourage frequent glucose monitoring, especially in populations that rarely monitor themselves otherwise. As such, it promises to improve patients' glycemic awareness and consequently their glycemic control and thus reduce diabetes-related complications.

## Figures and Tables

**Table 1:** Patient characteristics and the number of paired GlucoTrack-invasive readings.

Category		Number of subjects	Number of paired readings
Gender	Male	91	4114
	Female	81	3597
Age (year)	18–60	87	3820
	>60	85	3891
Body mass (kg)	<75	51	2260
	75–90	63	2825
	>90	58	2626
Ear piercing	Yes	75	3329
	No	97	4382
All		172	7711
